# Quad-Element Implantable MIMO Antenna for Wireless Capsule Endoscopy

**DOI:** 10.3390/s26072276

**Published:** 2026-04-07

**Authors:** Amor Smida, Jun Jiat Tiang, Mohamed I. Waly, Surajo Muhammad

**Affiliations:** 1 Department of Medical Equipment Technology, College of Applied Medical Science, Majmaah University, Al-Majmaah 11952, Saudi Arabia; m.waly@mu.edu.sa; 2 Centre for Wireless Technology, CoE for Intelligent Network, Faculty of Artificial Intelligence and Engineering, Multimedia University, Cyberjaya 63100, Selangor, Malaysia; 3 Center for Intelligent Network Telekom Research and Development (TM R&D), Sdn Bhd, Multimedia University, TM Innovation Centre, Lingkaran Teknokrat Timur, Cyberjaya 63100, Selangor, Malaysia

**Keywords:** antenna, bandwidth, channel capacity, ECC, diversity gain

## Abstract

Compared to antennas bearing a single port, MIMO antennas with several ports enable higher data throughput by exploiting spatial diversity. This capability is essential for next-generation implantable medical devices, where high channel capacity is a key requirement. A quad-element implantable MIMO antenna is designed and practically validated at 1420 MHz in this paper. It occupies a compact volume of $7\times 8\times 0.1\;{\mathrm{mm}}^{3}$ ($5.6\;{\mathrm{mm}}^{3}$). The compactness is realized by combining high-permittivity substrate (Rogers 3010 with relative permittivity of 10.2) with meandered radiator paths, which increase the effective current length while maintaining a small physical size. All antennas have very small mutual coupling with isolation of more than 31.78 dB, which is mainly due to the spacing of 1 mm between the elements and the substrate, which is thin. The peak realized gain for each antenna element is −27.3 dBi. The simulation is performed within a capsule-like structure, which is embedded in the stomach tissue model. The experimental verification is carried out by embedding antenna within minced meat. The ECC, channel capacity, and link margin are also evaluated and found to be satisfactory. The proposed antenna ensures reliable communication performance, with the transmission range being as high as 2.5 m, link margin being 15 dB, and the data rate being 120 Mb/s. The proposed antenna ensures a good level of ECC, which is less than 0.1. The SAR is 52.3 W/kg at 1420 MHz. This design is favorable for implants because of the small size, good impedance matching, high isolation, low correlation, good level of gain, and good link performance.

## 1. Introduction

Wireless patient monitoring systems have a critical role in modern healthcare by enabling continuous, safe, and remote observation of patients [1,2,3]. These platforms reduce repeated hospital visits needed for routine checkups, diagnosis, and data acquisition [4,5]. A central part of such systems is the medical implantable device (MID), which senses physiological parameters and wirelessly transfers data, images, and videos [6,7]. In recent years, MIDs have been explored for cardiac pacemakers [8], neural recording and stimulation systems [9], wireless capsule endoscopy [10], and intraocular pressure monitoring for glaucoma [11,12,13]. These devices are very useful for identifying any issues within the body and provide information for early diagnostics. Implantable antenna design has become an active research topic [14,15].

An implantable antenna must operate properly while remaining compatible with a capsule or flat implant that contains additional electronic parts within a millimeter-scale volume. This requirement places strict limits on antenna size, radiation efficiency, and communication reliability. Achieving miniaturization without causing unacceptable performance degradation remains difficult. Some of the methods proposed for this purpose are the employment of high-permittivity substrate [16], meandered radiators, slow wave concepts [17], and reactive loading [18]. These methods work well in bringing the frequency down and maintain other attributes unaffected. In addition to this, the wireless link between the implant and the on-body receiver is another challenge. Reported implantable antennas cover single- [19,20], two- [21], three- [22], and four-band [23] operation. In a SISO implantable antenna, the achievable data rate remains constrained once the antenna structure and available bandwidth are fixed. In addition, SISO links are more sensitive to multipath fading and provide lower spectral efficiency, which restricts throughput [24,25].

These limitations have motivated the adoption of multiple port techniques in implantable and biomedical channel links [26,27]. By exploiting multipath propagation, MIMO antennas can improve robustness and spectral efficiency relative to SISO systems, leading to higher data throughput [28]. This feature is particularly useful for implantable scenarios because the surrounding tissues strongly influence the communication channel [29,30,31,32]. Several implantable systems based on MIMO operation have, therefore, been reported [33,34,35,36]. In [33], a compact implantable MIMO structure used a neutralization line to weaken inter-element coupling and improve isolation for high-speed telemetry. In [34], a four-element implanted MIMO antenna employed vias to suppress coupling and broaden the operating bandwidth. Another reported direction is to preserve a very compact implant while pursuing more isotropic radiation rather than relying only on an array arrangement. That approach can be valuable for monitoring systems and implants placed in biologically sensitive regions with comparatively less conductivity. When electromagnetic properties of these regions are properly considered and coupling is controlled, telemetry with high speed can be realized. These features make such designs vital for patient monitoring and other healthcare links that require dependable wireless transfer. Other implantable MIMO antennas have also appeared in the literature. For example, ref. [37] used electromagnetic bandgap (EBG) structures at 2.45 GHz to improve isolation. A wideband implantable MIMO antenna was presented in [38], an antenna based on radiators was reported in [39], and a miniaturized MIMO antenna based on dual meandered resonators was described in [40]. Despite these advances, implantable MIMO antennas still face two main issues: increasing the number of elements usually enlarges the implant size, and close element spacing can produce strong mutual coupling that degrades MIMO channel performance. Accordingly, a practical implantable MIMO antenna must remain small while preserving strong electrical performance.

To address these requirements, this work presents a small-sized quad-port implantable antenna with high isolation for biomedical links supporting high-speed telemetry. The design uses a meandered resonator together with a high-permittivity substrate to realize miniaturization. High isolation is obtained through the combination of a very thin substrate and carefully selected spacing between the radiators. The final antenna occupies $7\times 8\times 0.1\;{\mathrm{mm}}^{3}$ ($5.6\;{\mathrm{mm}}^{3}$) and provides 31.78 dB isolation at 1.42 GHz. The antenna system is composed of four radiating monopoles arranged with shared ground and 1 mm separation between adjacent elements. To assess safety and communication quality, SAR and wireless link metrics are evaluated. They indicate low inter-element correlation and stable high-data-rate performance, which supports this antenna for modern medical systems. The main contribution and novelty of this work is the design of a highly compact implantable MIMO antenna that achieves high inter-element isolation despite its small footprint.

The remainder of the paper is organized as follows: Section 2 describes the antenna design methodology and evolution stages. Section 3 presents the simulation and experimental results, including S-parameters, current distribution, SAR, radiation patterns, link margin, and MIMO performance metrics. Section 4 compares the proposed design with state-of-the-art works, and Section 5 concludes the paper.

## 2. Design Methodology

**Figure 1:**
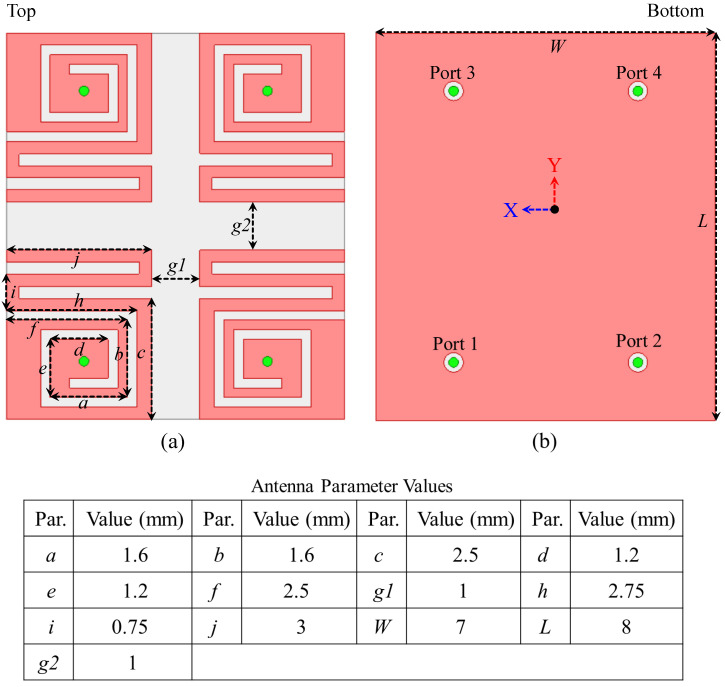
Final and optimized four-element MIMO antenna and their dimensions (**a**) Top view, and (**b**) bottom view.

The physical layout of antenna as well as corresponding dimensional parameters are presented in Figure 1. The implantable MIMO configuration is formed by four compact radiating elements based on meandered resonator paths. Each radiator is fed separately through coaxial probe bearing impedance of 50 ohm. To enlarge effective electrical length inside a tightly constrained implantable volume, configuration of meandered structure is opted. At the same time, the combination of inter-element spacing and an ultra-thin substrate reduces electromagnetic coupling and helps maintain high isolation among the four ports.

The radiators and common ground are carefully structured over substrate of Rogers RO3010 of 10.2 permittivity. This substrate is 0.1 mm thick. The copper thickness on both the top and bottom sides is 17 µm. The full antenna occupies only $7\times 8\times 0.1\;{\mathrm{mm}}^{3}$, or $5.6\;{\mathrm{mm}}^{3}$. The thin substrate is deliberately chosen because it keeps the fields confined close to the antenna and reduces surface-wave propagation between nearby elements. Although implantable antennas are often studied in simplified single-layer, multilayer, or phantom environments, an actual biomedical implant operates as part of a complete device. For that reason, this radiating structure is designed in the presence of a dummy implant platform rather than as an isolated radiator. Depending on the target organ and implantation depth, the device form can vary; however, a capsule-type format is widely utilized in WCE operation.

**Figure 2:**
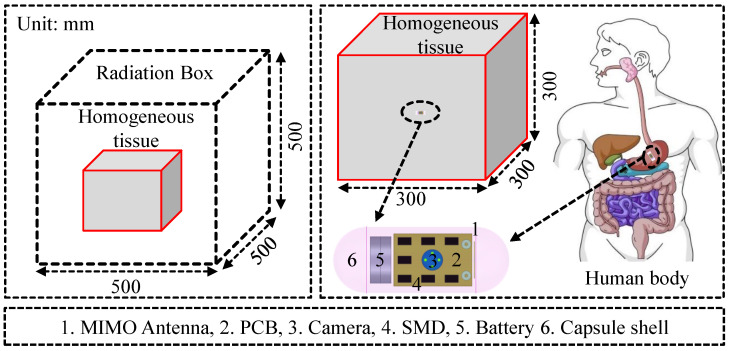
Antenna location within the human body model used for analysis and configuration of the implantable device showing electronic arrangement.

This design is, therefore, integrated into a capsule-shaped implant (Figure 2). In addition, the capsule contains the wireless power transfer receiver and other necessary parts of the implant. This is to clarify that these parts are dummy rather than exact parts. These parts are enclosed by an PLA shell. The complete device measures 23 mm in length and 12 mm in diameter, which represents a practical form factor for deep-tissue implantable biomedical devices.

### 2.1. Simulation Environment

Implantable antennas must be analyzed differently from free-space antennas because biological tissues are lossy and dispersive. For this reason, performance is usually evaluated inside tissue-equivalent media or anatomical phantoms. In the present study, the initial design stage places the antenna inside a homogeneous stomach-tissue block measuring $300\times 300\times 300\;{\mathrm{mm}}^{3}$, as indicated in Figure 2. The tissue model is assigned frequency-dependent material parameters. For 1420 MHz, relative permittivity and conductivity are taken as $\varepsilon_{r}=63.9$ and σ=1.45 S/m, respectively, based on reported tissue data [41]. Since stomach-tissue properties vary with frequency, the antenna response is affected by the values listed in Table 1. We have used ANSYS HFSS v23 for all simulations [42]. For the first stage, the mesh size was 8,031,050 elements with a simulation time of approximately 1 h 29 min. For the second stage, the mesh size was 9,300,072 elements with a simulation time of approximately 1 h 40 min. For the third stage, the mesh size was 11,405,014 elements with a simulation time of approximately 1 h 58 min. For the fourth stage, the mesh size was 13,671,000 elements with a simulation time of approximately 2 h 35 min.

The standalone antenna is first optimized while embedded 50 mm deep inside the homogeneous tissue block, without the capsule enclosure. This step establishes the baseline electromagnetic behavior under controlled conditions. Afterward, the same design is positioned in device and optimized again in stomach-tissue environment so that coupling between the antenna, the shell, and the internal components is included. A simplified cubical tissue model is used during the earlier stages to reduce computational cost. Once the design of the combined antenna capsule is fixed, the implant is positioned in abdominal region (phantom) to validate its performance under more realistic conditions.

For all cases, the tissue model or phantom is surrounded by a box (sides of the box are assigned as radiation boundaries), as shown in Figure 2. Terminal solver with an interpolating frequency sweep is used. The solution frequency is fixed at 1.42 GHz, and the maximum adaptive passes are set to 19. The convergence is limited to a maximum ΔS of 0.002. In addition, a frequency sweep from 500 MHz to 5000 MHz is performed to analyze the whole antenna characteristics in the desired frequency range.

### 2.2. Design Evolution Stages for Unit Element

**Figure 3:**
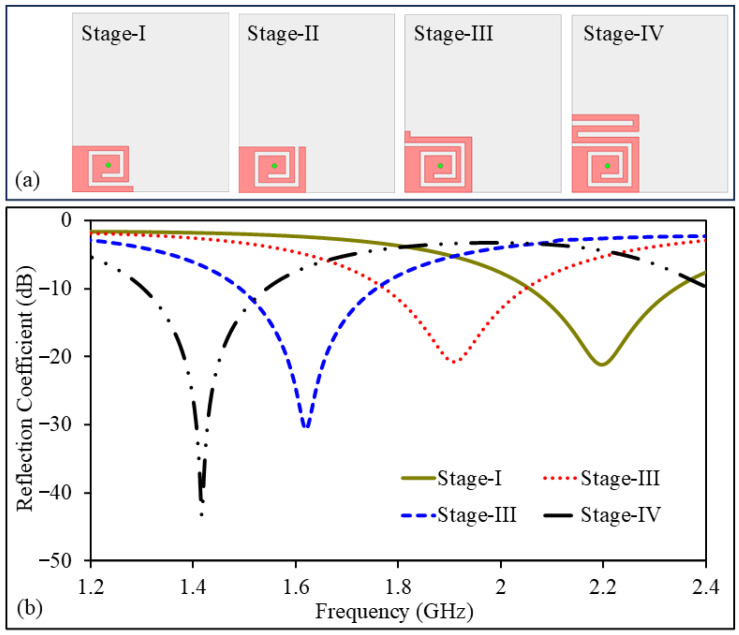
(**a**) Illustration of design stages. (**b**) Reflection coefficients during four optimization stages.

The final configuration of the proposed antenna design will comprise four identical radiating elements. Before proceeding with practical verification of design, extensive simulations are performed to optimize the design for superior electrical performance. The design process is carried out in a systematic manner, starting by a unit radiating part and ending on MIMO design. Intermediate optimization steps of the design process are illustrated in Figure 3. After the unit design is optimized for desired performance requirements, four identical elements are combined to form the MIMO antenna design. In this process, critical design parameters are adjusted.

The primary design goals are to achieve good impedance matching for both ports ($S_{11}$ and $S_{22}$) across the intended operating band, obtain sufficient impedance bandwidth, and minimize coupling. Key design stages leading to the final design are described below.

**Stage I:** Dielectric laminate fully backed by a copper on top and ground plane is initially designed. A compact spiral–meandered design is then etched on the dielectric laminate. The design is excited using a coaxial probe feed. This geometry is selected because it enables a long effective current path to be accommodated within a limited physical area, maintaining compact dimensions. The initial radiator length is estimated using the quarter-wavelength approximation

(1) $$L_{\mathrm{res}}=\frac{c}{4f\sqrt{\varepsilon_{r}}},$$

where $L_{\mathrm{res}}$ represents the effective resonator length, *c* is the speed of light, *f* is the operating frequency, and $\varepsilon_{r}$ is the relative permittivity of the substrate. In the first iteration, the antenna exhibits a resonance around 2.2 GHz with acceptable impedance matching.

**Stage II:** In the second stage, additional meandered sections are introduced at the open end of the radiator. These sections extend the effective electrical length without increasing the physical footprint. As a result, the resonant frequency shifts toward a lower value. The closely spaced meandered traces also introduce extra capacitive coupling, which further contributes to resonance reduction. The impact of this added reactance can be understood through the slow-wave concept, expressed as

(2) $$v_{p}=\frac{1}{\sqrt{L_{\mathrm{res}}C_{\mathrm{res}}}}=\frac{v_{c}}{\sqrt{\varepsilon_{\mathrm{eff}}}}=f\lambda_{g},$$

where $v_{p}$ is the phase velocity, $L_{\mathrm{res}}$ and $C_{\mathrm{res}}$ denote the effective inductive and capacitive components of the radiator, respectively, and $\varepsilon_{\mathrm{eff}}$ is the effective permittivity. Increasing either the effective inductance or capacitance reduces the phase velocity, leading to a lower resonant frequency. At this stage, the antenna resonance shifts to approximately 1.9 GHz, with a reflection coefficient of about −20.8 dB.

**Stage III:** In the third iteration, another meandered section is introduced into the radiator. This increases the electrical length of the radiator even more, as well as the capacitive loading effect of the radiator. Due to the inverse relationship between the resonant frequency and the effective length, the frequency at this point decreases even more. At this point, the frequency at which the antenna operates is approximately 1.62 GHz.

**Stage IV:** In the final optimization step, the radiator length is fine-tuned to accurately position the resonance at 1.42 GHz. The optimized single-element antenna provides an impedance bandwidth of 242 MHz, ranging from 1302 to 1544 MHz, with a minimum reflection coefficient of approximately −43 dB. The optimized single element of the antenna has realized gain of −23.6 dBi and total efficiency of −26.3 dB at 1420 MHz. Such low gain and efficiency are common for implantable antennas because of strong losses and power absorption in surrounding biological tissues. Finally, having completed the single-element antenna, the entire MIMO antenna system is constructed. To minimize the effect of mutual coupling, an ultra-thin substrate is employed, with all adjacent radiators having a uniform edge-to-edge separation of 1 mm. This method proves beneficial, especially in small implantable devices, as a majority of the components are packed tightly together. In a dense arrangement, the surface currents are able to spread over the common ground, thus affecting the radiation characteristics. However, with the currents confined to the substrate, along with a suitable spacing, the mutual coupling effect is reduced. This method proves suitable for a biomedical application, as the device is small, simple, and biocompatible.

### 2.3. Impact of Batteries on the Antenna Performance

To investigate the impact of batteries on the antenna performance, we have carried out simulations with and without adding batteries in the capsule. It was observed that the S-parameters of the antenna remain unchanged, and slight changes in gain and efficiency were observed. With battery, the gain and efficiency were decreased by 0.3 dBi and 0.4 dB at 1420 MHz. This is due to the fact that the antenna is mounted at one end of the capsule, while the battery is placed at the opposite end. This separation reduces direct electromagnetic interaction. As a result, the battery slightly impacts gain and efficiency, while the S-parameters remain unchanged. The summary is shown in Table 2.

## 3. Simulation and Experimental Results

**Figure 4:**
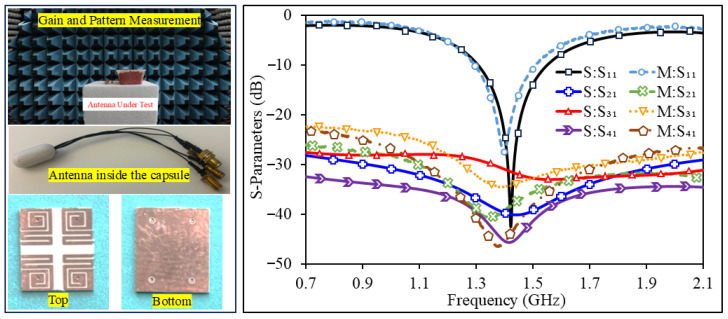
Simulated and measured S-parameters.

The fabricated antenna (on Rogers RO3010) occupying $7\times 8\times 0.1\;{\mathrm{mm}}^{3}$ is shown in Figure 4. Before fabrication, the design is verified through full-wave simulations using the three-dimensional electromagnetic solver Ansys HFSS. The radiating traces and ground plane are produced by standard chemical etching on the substrate. To reproduce a practical implant environment during measurements, a capsule enclosure is fabricated by three-dimensional printing. The shell is made of PLA. For impedance measurements under implant-like conditions, the capsule-loaded antenna is inserted into minced meat used as a tissue-equivalent medium.

### 3.1. Simulated S-Parameters

After optimization steps, the antenna resonates at 1420 MHz and provides an impedance bandwidth (BW) of 242 MHz. Figure 4 presents the simulated scattering parameters. Across the operating band, isolation between any two ports stays better than 29.47 dB and reaches 31.78 dB at the center frequency. Because of the geometric symmetry of the layout, $S_{22}$, $S_{33}$, and $S_{44}$ follow the same response as $S_{11}$, so only $S_{11}$ is plotted for simplicity. The obtained bandwidth fully covers the targeted Wireless Medical Telemetry Service (WMTS) band around 1.42 GHz. Since biological tissues have frequency-dependent dielectric properties that vary from one anatomical region to another, resonance detuning can occur after implantation. The wide bandwidth helps the antenna keep working even if the frequency changes slightly. This makes the system more reliable in different implantation conditions.

### 3.2. Results of Fabricated Antenna

Finally, optimized design is realized for experimental verification. In the experiment, the prototype is integrated with a dummy device in meat to mimic the in-body environment. The proposed prototype has resonance at 1398 MHz with a 10 dB BW of 216 MHz from 1296 to 1512 MHz. The isolation is higher than 30.2 dB in the band of operation. This is a relatively wider bandwidth that can cover the frequency shift caused by the change in tissue properties. The simulated results very well matched with fabricated data, proving the effectiveness of the proposed design.

### 3.3. Current Distribution

The proposed MIMO system places four radiators at the substrate corners facing one another. For proper MIMO behavior, each element should operate with minimal interference from the others. Lower interaction between elements generally leads to better overall system efficiency and reliability. One common way to inspect coupling is to excite one port and observe the current induced on the remaining passive elements. In an ideal case, the passive radiators would carry no current, although in practice a small amount of leakage is always present.

**Figure 5:**
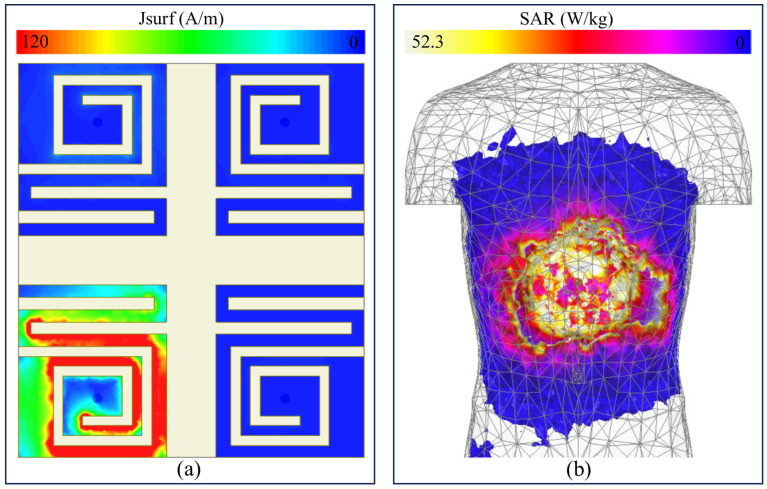
For 1420 MHz: (**a**) current density plot and (**b**) 10 g SAR.

In this study, Port-1 (element 1) is activated, and the current plot is examined at 1420 MHz in Figure 5a. Most of the current remains concentrated on the driven resonator, whereas only weak induced current is visible on the passive elements. This confirms that inter-element coupling is low, which is consistent with the isolation results obtained from the scattering parameters.

### 3.4. SAR Analysis at 1420 MHz

The specific absorption rate (SAR) quantifies how much electromagnetic energy is absorbed by tissue when the implantable antenna is active. According to IEEE C95.1-1999, a SAR of 10 g must remain below 2 W/kg. SAR evaluation is, therefore, required to determine whether the implant can operate within the accepted exposure limits [43].

Here, SAR is evaluated in a heterogeneous torso representing the stomach region. During the calculation, one antenna port at a time is active to determine maximum absorbed energy in the nearby tissues. Figure 5b shows the normalized SAR distribution for an implantation depth of 50 mm, which is the same depth used in the homogeneous stomach model. The SAR of the antenna was initially found to be 52.3 W/kg at 1 W input power. For implantable devices, the maximum allowable transmit power is −16 dBm (25 µW). Since SAR scales linearly with input power, the value is normalized to this power level based on the 10 g averaging convention. The resulting SAR is approximately 0.0013 W/kg, which is significantly below the IEEE C95.1-2019 safety limit of 2 W/kg (10 g). Therefore, the proposed design is safe for implantable applications. The result is presented at 1420 MHz with a single port excited. The highest SAR appears in the tissue immediately surrounding the antenna, where the electromagnetic fields are strongest. As the wave propagates through tissue, the absorbed power decreases with distance. At 1420 MHz, the comparatively longer wavelength allows the fields to extend more deeply into the surrounding tissue, which produces a wider SAR distribution around the implant site. Even so, the peak absorption remains localized near the antenna, while tissues farther away experience much lower exposure.

### 3.5. Far-Field Patterns

**Figure 6:**
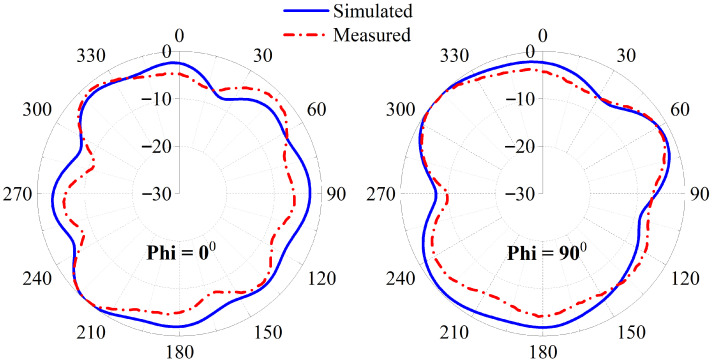
Two-dimensional patterns at 1420 MHz.

Radiation patterns are characterized through far-field measurements in an anechoic chamber. During each measurement, one element is sourced and the remaining connected to matched loads so that reflections and unwanted coupling are minimized. The measured patterns are presented in Figure 6. Such a pattern is beneficial for wireless capsule implants because the capsule can translate and rotate unpredictably inside the gastrointestinal tract. An omnidirectional antenna, therefore, helps preserve the wireless link regardless of the capsule orientation and supports stable data transfer.

At 1420 MHz, the peak gain obtained from simulation is −27.3 dBi, whereas the measured peak gain is −26.01 dBi. The small difference between these values shows good agreement between simulation and experiment and supports the accuracy of the proposed design. This antenna has total efficiency of −27.1 dB.

### 3.6. Link Margin Calculations

This design targets implantable applications that require dependable high-data-rate communication. For this reason, link budget analysis is used to verify whether the receiver can obtain enough power for stable operation. In practical wireless systems, extra margin is usually included to improve reliability. In this work, a minimum link margin of 10 dB is assumed.

The link margin can be expressed as

(3) $$LM\;\left[\mathrm{dB}\right]=P_{a}-P_{r}$$

where $P_{a}$ represents the available power at the receiver, and $P_{r}$ denotes the minimum required power for successful data decoding. These quantities are calculated as

(4) $$P_{a}\;\left[\mathrm{dB}\right]=P_{t}+G_{t}+G_{r}-L_{f}-N_{\circ }$$

(5) $$P_{r}\;\left[\mathrm{dB}\right]=\frac{E_{b}}{N_{\circ }}+10\log_{10}\left(B_{r}\right)-G_{c}+G_{d}$$

In these expressions, $P_{t}$ is the transmitted power, $G_{t}$ and $G_{r}$ denote the gains of the transmitting and receiving antennas, and $L_{f}$ is the free-space path loss. The noise spectral density is represented by $N_{\circ }$. The free-space path loss is written as

(6) $$L_{f}\;\left[\mathrm{dB}\right]=10\log_{10}{\left(\frac{4\pi d}{\lambda }\right)}^{2}$$

and the noise spectral density is obtained from

(7) $$N_{\circ }=10\log_{10}\left(kT_{\circ }\right)$$

where *k* is Boltzmann’s constant, and $T_{\circ }$ is the absolute temperature.

For the calculations, the receiving antenna is assumed to be an ideal half-wavelength dipole with a gain of 2 dBi. The separation between the implanted transmitter and the external receiver is represented by *d*. To simplify the analysis, polarization mismatch and impedance mismatch losses are neglected. The remaining link parameters follow reported values from earlier studies [43].

**Figure 7:**
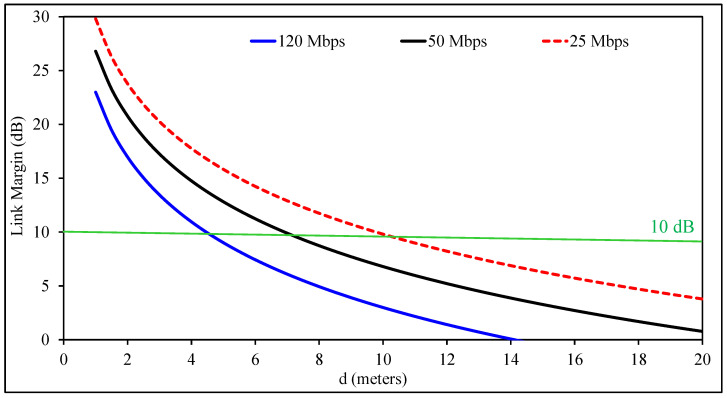
Estimated wireless link margin at 1420 MHz.

Figure 7 shows the resulting link margin. Communication becomes possible when the link margin is above 0 dB; however, a design target of at least 10 dB is adopted here for stable and low-error operation. In this work, a design margin of 10 dB is adopted to account for unknown and unmodeled losses, including capsule rotation, tissue inhomogeneity, and additional propagation uncertainties. A relatively small transmitted power of $P_{t}$ = 25 µW is used in the evaluation to reduce possible interference with nearby wireless systems.

The results show that the achievable communication range decreases when the data rate increases, which is expected because higher throughput requires a higher signal-to-noise ratio at the receiver. Even with this tradeoff, the proposed system can sustain communication over distances approaching 5 m while supporting a data rate of 120 Mb/s. This makes the antenna suitable for applications requiring high-throughput wireless links, including capsule endoscopy, minimally invasive procedures, and other advanced biomedical monitoring systems.

### 3.7. MIMO Channel Capacity

The main advantage of a multiple-input, multiple-output (MIMO) antenna system is that it can raise data throughput without requiring more bandwidth or higher transmit power. If all propagation channels were fully independent, the channel capacity would increase with the number of transmitting and receiving antennas. In practice, however, the channels are partly correlated because of mutual coupling, limited spacing, and propagation effects. As a result, channel capacity depends not only on the number of antenna elements but also on the level of correlation between the channels. Low-correlation and high-isolation elements are, therefore, required to approach the theoretical MIMO limit. For an N×N MIMO system, the channel capacity can be expressed as [44]

(8) $$CC=\log_{2}\left(\det\left[I+\frac{SNR}{N}HH^{*}\right]\right)$$

where CC represents the channel capacity, *I* is the identity matrix, SNR denotes the signal-to-noise ratio, *H* is the channel matrix containing the amplitude and phase information of the antenna channels, and $H^{*}$ is the conjugate transpose of *H*.

Channel capacity may be obtained either from measurements in realistic propagation environments or from analytical models based on the antenna radiation characteristics. Reverberation chambers are often used for this purpose because they recreate rich multipath conditions similar to indoor and outdoor propagation environments and enable statistical characterization without performing field measurements.

Another practical method derives the channel matrix from the measured or simulated radiation patterns. Following [45], the channel matrix *H* is written as

(9) $$H=\sqrt{\Psi_{t}}G\sqrt{\Psi_{r}}$$

where *G* is a random matrix with Gaussian-distributed entries, $\Psi_{t}$ represents the transmit correlation matrix, and $\Psi_{r}$ denotes the receive correlation matrix. These matrices describe the spatial correlation between the antenna elements and are obtained from the radiation patterns. The correlation coefficient between elements *x* and *y* is given by

(10) $$\Psi_{x,y}=\frac{\mu_{xy}}{\sqrt{\mu_{xx}\mu_{yy}}}$$

The term $\mu_{x,y}$ can be obtained from the two-dimensional radiation patterns of antenna *x* and antenna *y* as

(11) $$\mu_{x,y}=\int_{0}^{2\pi }\left[XPD\;A_{x,\theta }A_{y,\theta }^{*}+A_{x,\phi }A_{y,\phi }^{*}\right]d\phi$$

where $A_{\theta }$ and $A_{\phi }$ represent the θ- and ϕ-polarized radiation components of the antennas, and XPD denotes the cross-polarization discrimination.

**Figure 8:**
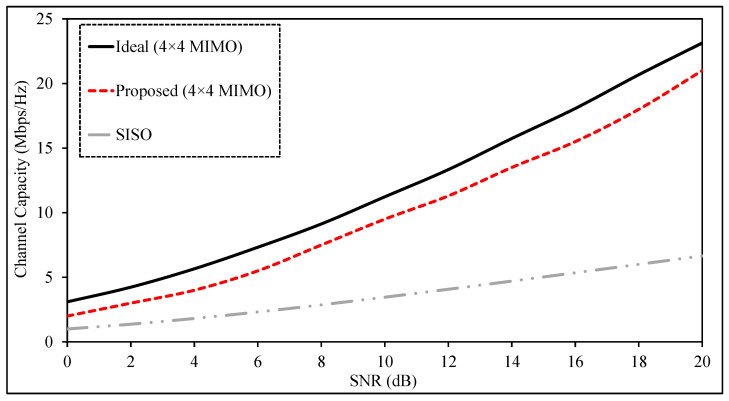
Estimated channel capacity of the wireless link operating at 1420 MHz.

The channel capacity of the proposed MIMO antenna system is obtained from the extracted two-dimensional radiation patterns. Figure 8 shows the channel capacity as a function of the signal-to-noise ratio (SNR). The proposed antenna performs between the two theoretical limits represented by an ideal SISO system and an ideal 4×4 MIMO system. The results confirm that the proposed design provides a clear capacity improvement over the SISO case, which supports its use in high-data-rate biomedical communication links.

### 3.8. Envelop Correlation Coefficient (ECC) and Diversity Gain (DG)

The ECC is a common metric used to judge the degree of dependence between antenna elements in a MIMO system. A value of zero represents completely independent channels. In practice, values below 0.5 are generally considered acceptable. ECC can be extracted either from scattering parameters or from far-field radiation patterns. In this work, it is obtained from the far-field patterns using (12) [26]. The calculated results indicate that the ECC stays below 0.1 throughout the operating band, including the resonance region. Such low values indicate strong diversity behavior and confirm the suitability of the antenna for high-data-rate implantable devices, including wireless capsule endoscopy.

(12) $$ECC=\frac{{\left|{\;\int \int \;}_{4\pi }\left(\vec{An_{i}}\left(\theta ,\phi \right)\cdot \vec{An_{j}}\left(\theta ,\phi \right)\right)d\Omega \right|}^{2}}{{\;\int \int \;}_{4\pi }{\left|\vec{An_{i}}\left(\theta ,\phi \right)\right|}^{2}d\Omega \;{\;\int \int \;}_{4\pi }{\left|\vec{An_{j}}\left(\theta ,\phi \right)\right|}^{2}d\Omega }$$

where $\vec{An_{i}}\left(\theta ,\phi \right)$ and $\vec{An_{j}}\left(\theta ,\phi \right)$ represent the three-dimensional radiation patterns of the *i*th and *j*th antenna elements, respectively, and Ω denotes the solid angle.

Diversity gain (DG) is another useful MIMO indicator because it reflects the improvement in signal reliability provided by diversity operation. For completely uncorrelated channels, the maximum DG is 10 dB. In this study, DG is calculated from (13) [26], which directly links DG to ECC. The results show that DG remains above 9.95 dB across the full operating band, confirming strong diversity performance for the proposed antenna system.

(13) $$DG=10\sqrt{1-{\left(ECC\right)}^{2}}$$

## 4. Comparison with Other Designs

Table 3 compares the proposed design with previously reported implantable antennas. Many of the earlier structures either require a larger physical volume or provide lower inter-element isolation. Some reported antennas also rely on a single element, which limits their ability to support true MIMO operation. In contrast, several multi-element designs improve channel behavior but do so at the cost of increased size (see Table 3).

The proposed antenna combines a compact volume of only $5.6\;{\mathrm{mm}}^{3}$ with a four-element configuration and an isolation level of 31.78 dB. This combination shows that the design achieves a favorable tradeoff between miniaturization and inter-element decoupling, which is important for implantable biomedical devices that demand reliable wireless communication within a very small footprint.

## 5. Conclusions

A compact quad-element implantable MIMO antenna for the WMTS band at 1420 MHz has been presented and experimentally verified. The proposed structure occupies $7\times 8\times 0.1\;{\mathrm{mm}}^{3}$ and achieves miniaturization by combining a high-permittivity substrate with meandered radiating paths. Despite the small size and only 1 mm separation between elements, the antenna maintains isolation greater than 31.78 dB. The peak realized gain of each element is −27.3 dBi. The design is studied in a stomach-tissue environment and validated experimentally using minced pork meat. MIMO performance is assessed through ECC, channel capacity, and link margin. The ECC remains below 0.1, indicating low correlation and effective diversity behavior. At an SNR of 20 dB, the channel capacity reaches 19.8 Mb/s/Hz. The link analysis indicates a communication range up to 2.5 m with a 15 dB link margin and support for a 120 Mb/s data rate. The calculated SAR at 1420 MHz is 52.3 W/kg. These results show that the proposed antenna provides reliable wireless operation in a compact form, making it a strong candidate for implantable biomedical applications such as advanced capsule endoscopy systems.

## Figures and Tables

**Table 1:** Dielectric parameters of stomach tissue at different frequencies, presenting the corresponding values.

Frequency (GHz)	1	1.15	1.3	1.45	1.6	1.75
Conductivity (S/m)	1.23	1.30	1.38	1.47	1.56	1.66
Relative permittivity	64.79	64.44	64.13	63.84	63.57	63.31
Loss tangent	0.34	0.31	0.29	0.28	0.27	0.26

**Table 2:** Antenna performance with and without batteries of capsule.

Parameter	With Batteries	Without Batteries
Resonant Frequency	1420 MHz	1420 MHz
Bandwidth	242 MHz	242 MHz
Gain	−27.3 dBi	−27 dBi
Total Efficiency	−27.1 dB	−26.7 dB

**Table 3:** Comparison with state-of-the-art implantable antennas.

Ref.	Size (mm^3^)	Substrate Relative Permittivity	No. of Elements	Antenna Profile	Frequency (MHz)	Isolation (dB)	Gain (dBi)
[30]	13.2	10.2	1	Planar	2450	—	−8.4
[31]	2.97	10.2	1	Planar	2450	—	−9.7
[32]	9.01	10.2	2	Planar	915	29.7	−24.6
[33]	70	10.2	2	Planar	2450	22.93	−32.15
[34]	23.6	10.2	4	Planar	433	32.6	−28.3
[35]	3375	2.2	4	Cubic	2400, 5800	32	−18.5
[36]	307.4	10.2	2	Planar	402	25.6	−26
[37]	434.6	10.2	4	Planar	2400	15.9	−15.18
This work	5.6	10.2	4	Planar	1420	31.78	−27.3

## Data Availability

The original contributions presented in this study are included in the article. Further inquiries can be directed to the corresponding authors.
